# Minimally Invasive Magnetic Removal of Forehead Foreign Body

**DOI:** 10.7759/cureus.25168

**Published:** 2022-05-20

**Authors:** Dieter Brummund, Angela Chang, Ricardo Castrellon

**Affiliations:** 1 Plastic Surgery, Larkin Community Hospital, Miami, USA; 2 Department of Anesthesiology, Aventura Hospital and Medical Center, Miami, USA; 3 Plastic Surgery, Larkin Community Hopistal, Miami, USA

**Keywords:** tissue expander, metallic foreign body, ferromagnetic, surgical magnet, non-powder firearm

## Abstract

Residual foreign bodies are common sequelae of penetrating injuries. These may be left in situ or extracted and can be difficult to localize, often requiring wide exposure, which can be difficult to achieve in cosmetically sensitive areas. Different technological adjuncts are used to facilitate foreign body removal including fluoroscopy, ultrasound, and more recently, surgical magnets. This case describes and illustrates the unusual technique of using a sterile surgical magnet to both localize and remove a foreign body in the head and neck region using a minimally invasively technique.

## Introduction

Non-powder firearm injuries are a common cause of trauma in the pediatric population. These include paintball guns, BB guns, pellet guns, and air rifles. The severity of the injury is associated with projectile velocity. Despite protective measures including patient education, marking of non-powder firearms with an orange barrel tip, and the use of protective eyewear, this continues to be a common cause of injury. The majority of these patients are males between the ages of six and 12. The most common non-powder firearm injury is a residual foreign body [1].

Residual foreign bodies may be left in situ or extracted but can be difficult to localize and often require wide exposure, which may be difficult to achieve in cosmetically sensitive areas such as the face. Different technological adjuncts used to facilitate foreign body removal include fluoroscopy, ultrasound, and more recently surgical magnets [2]. This case describes and illustrates the unusual yet simple technique of using a sterile surgical magnet to both localize and remove a foreign body in the head and neck region.

## Case presentation

A 13-year-old male presented with a painless forehead mass following an accidental self-inflicted non-powder gunshot wound with a BB gun 12 weeks prior. Physical examination revealed a 2 cm mobile mass in the right paramedian supraorbital region that was found to be ferromagnetic. Plain films demonstrated a radiopaque object in the subcutaneous tissue overlying the frontal bone. The patient and family opted for foreign body removal under local anesthesia.

The areas surrounding and deep to the forehead mass were infiltrated with 1% lidocaine with epinephrine to numb the operative site and hydrodissect the foreign body and capsule away from deeper structures. A 5 mm horizontal incision was made in an overlying forehead rhytid. A sterile magnetic port finder (Sientra) was utilized to localize and extract the metallic object (Video 1).

**Video 1:** Minimally Invasive Magnetic Forehead Foreign Body Removal Note minimally invasive approach and magnetic removal of the mass.

Once extracted, the ferromagnetic nature of the excised fragment was then reconfirmed with the surgical magnet (Video 2).

**Video 2:** Ferromagnetic Foreign Body Ferromagnetic nature of foreign body confirmed ex-vivo.

**Figure 1:**
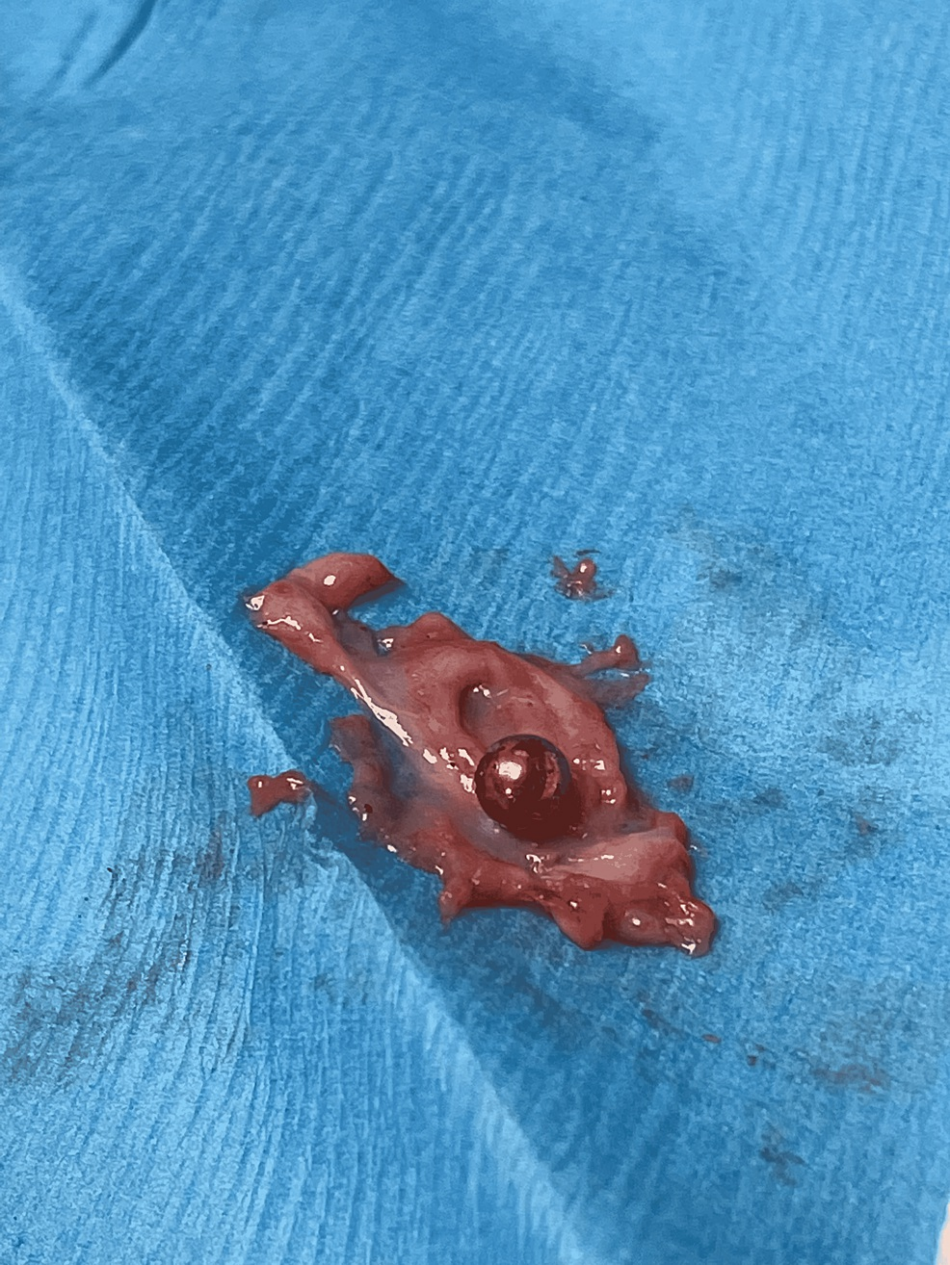
Retained BB projectile with surrounding capsule Note copper coating and thick surrounding capsule.

The capsule was opened revealing a metallic BB projectile (Figure 1).

Following removal of the foreign body, the surrounding capsule was excised to prevent postoperative seroma and recurrence of the mass. The wound was loosely approximated with a dissolvable suture.

The patient tolerated the procedure well. On one month follow-up, the mass had not recurred. 

## Discussion

Surgical magnets attract cobalt-, iron-, nickel-containing objects through the property of ferromagnetism. Contemporary magnets are composed of lightweight rare earth metals with a magnetic strength of 1.5 Tesla or 15,000 gauss, which is eight to 10 times the strength of older horseshoe magnets and 40-50 times the strength of common refrigerator magnets [3]. Surgical magnets are low-cost and are able to localize objects that are non-palpable and lack characteristic ultrasonographic features such as shadowing or the ring sign. They also avoid the risk of ionizing radiation associated with fluoroscopy [4].

Metallic foreign bodies may be left in situ if asymptomatic or extracted. Copper may generate an exaggerated local inflammatory response. Lead may leach into the body and cause systemic effects [4]. Objects may also migrate over time, further necessitating their removal. Indications for the removal of metallic foreign bodies include chronic pain, neuropathy, compression of neurovascular structures, granulomas, infection, and systemic toxicity [5]. In this case, the mass was growing and becoming cosmetically distressing to the patient, both of which are indications for removal.

Foreign bodies may be difficult to localize and often require wide exposure to successfully remove. Sufficient exposure may be difficult to achieve in cosmetically sensitive areas such as the face. Sterile surgical magnets have been used for decades in areas including the superficial soft tissues of the body, the ocular globe, and abdominal viscera, however, their use in the head and neck has only been reported more recently. This may be due to the unpredictable movement of the foreign body by the magnet during the extraction process, which can cause damage to adjacent neurovascular structures that are particularly abundant in the head and neck region [6]. 

From a technical standpoint, the property of ferromagnetism can be utilized to both localize and extract the foreign body. A hanging magnet is moved across the skin until the point is found at which it becomes perpendicular to the underlying foreign body, thus localizing the position [7]. A small incision can then be made and the magnet re-introduced to pull the foreign body out [8]. Spontaneous extraction of a ferromagnetic object has even been described from the force of the magnetic field immediately following an injury [9]. In this case, a sterile Sientra magnetic port locating device was utilized. The device consists of a series of two small magnets connected together to locate the drain and fill ports on the Sientra AlloX tissue expander for percutaneous filling. The Sientra magnet was utilized in this case due to its small size to assist with localization and extraction, while not blocking the operating field of view.

A minimally invasive approach using the sterile surgical magnet was used to both localize and extract a ferromagnetic foreign body in the forehead of this patient. This technique offered the best result given the cosmetically conspicuous location of the foreign body. The simple procedure was performed in the outpatient setting and the patient was very satisfied with the results. While useful in this instance, additional investigation is needed to further clarify which patients may most benefit from this approach. 

## Conclusions

Sterile surgical magnets can be used to localize and extract ferromagnetic foreign bodies. They are easy to use, available at a low cost, and avoid the risks and pitfalls of other surgical adjuncts commonly used in foreign body removal. This case demonstrates the simple yet unusual technique of using a surgical magnet to remove a retained BB pellet in the forehead. This technique should be further refined and standardized for use in patients with small superficial ferromagnetic foreign bodies to minimize the morbidity associated with other techniques.
